# Impaired cardiac contractile function in arginine:glycine amidinotransferase knockout mice devoid of creatine is rescued by homoarginine but not creatine

**DOI:** 10.1093/cvr/cvx242

**Published:** 2017-12-11

**Authors:** Kiterie M E Faller, Dorothee Atzler, Debra J McAndrew, Sevasti Zervou, Hannah J Whittington, Jillian N Simon, Dunja Aksentijevic, Michiel ten Hove, Chi-un Choe, Dirk Isbrandt, Barbara Casadei, Jurgen E Schneider, Stefan Neubauer, Craig A Lygate

**Affiliations:** 1 Division of Cardiovascular Medicine, Radcliffe Department of Medicine, BHF Centre of Research Excellence at the University of Oxford and the Wellcome Trust Centre for Human Genetics, Roosevelt Drive, Oxford OX3 7BN, UK;; 2 German Centre for Cardiovascular Research (DZHK), Partner Site Munich Heart Alliance, Institute for Cardiovascular Prevention (IPEK), Pettenkoferstraße 8a & 9, 80336 Munich, Germany;; 3 Walther-Straub Institute of Pharmacology and Toxicology, Ludwig Maximilians University, Goethestrasse 33, 80336 Munich, Germany;; 4 Department of Neurology, University Medical Center Hamburg-Eppendorf, Martinistrasse 52, 20246 Hamburg, Germany;; 5 Experimental Neurophysiology, German Center for Neurodegenerative Diseases (DZNE), 53175 Bonn, Germany;; 6 The Institute for Molecular and Behavioral Neuroscience, University of Cologne, Kerpener Str. 62, 50937 Cologne, Germany;; 7 Leeds Institute of Cardiovascular and Metabolic Medicine, University of Leeds, Leeds, LS2 9JT, UK

**Keywords:** Creatine kinase, Cardiac energetics, Contractile function, Homoarginine, AGAT

## Abstract

**Aims:**

Creatine buffers cellular adenosine triphosphate (ATP) via the creatine kinase reaction. Creatine levels are reduced in heart failure, but their contribution to pathophysiology is unclear. Arginine:glycine amidinotransferase (AGAT) in the kidney catalyses both the first step in creatine biosynthesis as well as homoarginine (HA) synthesis. AGAT^-/-^ mice fed a creatine-free diet have a whole body creatine-deficiency. We hypothesized that AGAT^-/-^ mice would develop cardiac dysfunction and rescue by dietary creatine would imply causality.

**Methods and results:**

Withdrawal of dietary creatine in AGAT^-/-^ mice provided an estimate of myocardial creatine efflux of ∼2.7%/day; however, *in vivo* cardiac function was maintained despite low levels of myocardial creatine. Using AGAT^-/-^ mice naïve to dietary creatine we confirmed absence of phosphocreatine in the heart, but crucially, ATP levels were unchanged. Potential compensatory adaptations were absent, AMPK was not activated and respiration in isolated mitochondria was normal. AGAT^-/-^ mice had rescuable changes in body water and organ weights suggesting a role for creatine as a compatible osmolyte. Creatine-naïve AGAT^-/-^ mice had haemodynamic impairment with low LV systolic pressure and reduced inotropy, lusitropy, and contractile reserve. Creatine supplementation only corrected systolic pressure despite normalization of myocardial creatine. AGAT^-/-^ mice had low plasma HA and supplementation completely rescued all other haemodynamic parameters. Contractile dysfunction in AGAT^-/-^ was confirmed in Langendorff perfused hearts and in creatine-replete isolated cardiomyocytes, indicating that HA is necessary for normal cardiac function.

**Conclusions:**

Our findings argue against low myocardial creatine *per se* as a major contributor to cardiac dysfunction. Conversely, we show that HA deficiency can impair cardiac function, which may explain why low HA is an independent risk factor for multiple cardiovascular diseases.

## 1. Introduction

The heart has a constant requirement for large amounts of energy in the form of adenosine triphosphate (ATP) and must maintain the ability to instantly respond to elevated workloads. Despite this, intracellular ATP levels do not change in the healthy heart, which implies a tight match between ATP demand and supply via glycolysis and oxidative phosphorylation.^1^ The creatine kinase (CK) phosphagen system is considered key to this by buffering ATP levels via rapid and reversible transfer of the phosphoryl group of ATP onto creatine (Cr) to form phosphocreatine (PCr)^2^: Cr + ATP ↔ PCr + ADP + H^+^.

In animal models and patients with heart failure, a decrease in total creatine levels and CK enzymatic activity is consistently observed, regardless of aetiology.^3^ For example, in patients with dilated cardiomyopathy, the ratio of PCr/ATP is reduced at an early stage of pathology and correlates with ejection fraction and survival.^4^ However, despite decades of research, the exact contribution (i.e. cause or effect) of reduced creatine to cardiac pathophysiology remains controversial.

Creatine is not synthesized in the cardiomyocyte, but must either be obtained from dietary sources or biosynthesized from arginine and glycine in a two-step reaction. The first, catalysed by arginine:glycine amidinotransferase (AGAT) pre-dominantly in the kidney, produces guanidinoacetate (GA), which is subsequently methylated by guanidinoacetate N-methyltransferase (GAMT), mostly in the liver, to form creatine. Creatine is subsequently absorbed by cardiomyocytes against a large concentration gradient via a specific creatine transporter (SLC6A8).^2^

Previous approaches have studied the effects of creatine depletion using creatine analogues such as guanidinopropionic acid (β-GPA), which competes with creatine for cellular uptake, but is a poor substrate for CK.^2^ Typically, baseline dysfunction is mild or absent, except at high workloads.^5–10^ The GAMT^-/-^ model, too, is in agreement with this general pattern, showing only a small reduction in left ventricular (LV) systolic pressure at baseline, but with impaired contractile reserve.^11^ The physiological relevance of this impairment is unclear since GAMT^-/-^ mice ran just as far and as fast as controls under voluntary and forced running protocols. Furthermore, following experimental myocardial infarction, cardiac function and remodelling were not altered in creatine-deficient GAMT^-/-^ mice.^12^

Both these approaches have major limitations. On the one hand, depletion of creatine by β-GPA is very slow, and there is always residual creatine (typically 10–50%). On the other hand, GAMT^-/-^ mice receiving a creatine-free diet are completely deficient in creatine, but accumulate millimolar concentrations of GA, which (as with β-GPA) can potentially compensate the creatine deficiency by participating in the CK reaction, albeit at a much reduced velocity.^13^ There is evidence that high levels of GA and β-GPA can be toxic or have off-target effects (e.g. inhibition of the Na^+^/K^+^ pump^14^), and it is therefore difficult to know with any certainty whether the observed effects are due to true creatine deficiency. For these reasons, the AGAT^-/-^ mouse has been keenly awaited in this field as a ‘pure’ model of creatine deficiency that might yield definitive answers. This is exemplified by the skeletal muscle phenotype, which is much more severe in AGAT^-/-^ than in GAMT^-/-^ mice and was completely reversed by creatine supplementation.^13^^,^^15^^,^^16^

It is known that the AGAT enzyme is also the principle source of homoarginine (HA) in both humans and mice.^17^^,^^18^ HA is a cationic amino acid structurally similar to arginine, but with an additional carbon in the alkyl chain.^19^ It has no established metabolic role and, until recently, was assumed to be an incidental by-product of the homologous urea cycle.^20^ Notably, low plasma HA levels have been associated with increased cardiovascular risk.^21^

Here, we present the first description of the cardiac phenotype in AGAT^-/-^ mice. Withdrawal of dietary creatine allowed us to estimate the rate of myocardial creatine efflux. We confirm that AGAT^-/-^ mice fed a creatine-free diet are devoid of creatine and PCr in the heart, and determined the effect on LV structure and function using MRI. Non-invasive magnetic resonance relaxometry allowed us to analyse body composition in detail and to demonstrate rescue by creatine supplementation. Finally, LV haemodynamic function was assessed under low-creatine conditions in the absence of potentially confounding guanidino compounds as well as after creatine and HA replenishment.

## 2. Methods

Detailed methods can be found in the Supplementary material online.

### 2.1 Animals and ethical statement

This investigation was approved by the Committee for Animal Care and Ethical Review at the University of Oxford and conforms to the UK Animals (Scientific Procedures) Act, 1986, incorporating Directive 2010/63/EU of the European Parliament. Arginine:glycine amidinotransferase knockout mice (AGAT^-/-^) have a homozygous knockout of the *Gatm^tm^*^1^^*.1Isb*^ allele created by homologous recombination.^22^ All procedures used mice aged 4–7 months on a pure C57BL/6J genetic background (backcrossed for >10 generations). Mice were housed in specific pathogen-free cages and maintained on a 12-h/12-h light–dark cycle under conditions of controlled temperature (20–22 °C) and humidity. Water and chow were available *ad libitum*.

### 2.2 Dietary manipulation of creatine and HA

**Figure 1:**
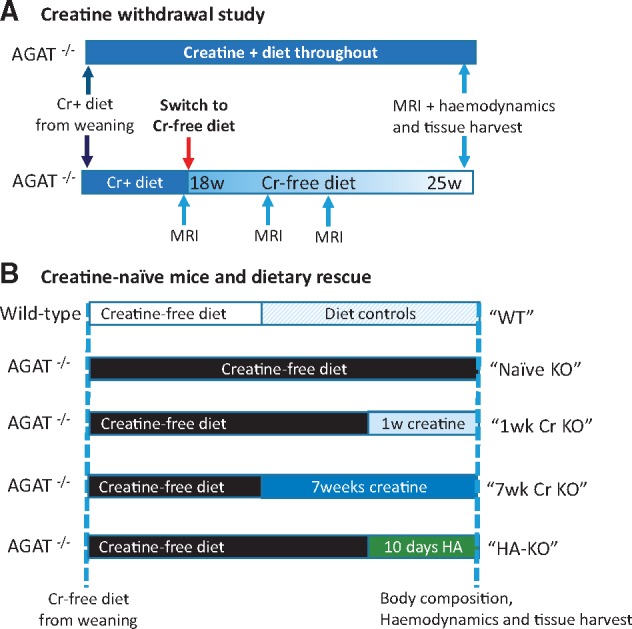
Schematic showing experimental study design. (*A*) Creatine withdrawal study: AGAT^-/-^ mice were bred and weaned onto a diet containing 0.5% creatine (w/w), which was then switched to a standard creatine-free diet at 18 weeks of age in the experimental group. These animals received multiple MRI and ^1^H-MRS examinations before LV haemodynamics and tissue harvest at ∼25 weeks of age. (*B*) Creatine-naïve mice and dietary rescue: WT and AGAT^-/-^ mice were bred and weaned onto a standard creatine-free diet for the first 4–5 months of life. Mice were then either maintained on this creatine-free diet, switched to a creatine supplemented diet for either 1 or 7 weeks, or given 14 mg/L of homoarginine for 10 days while maintaining a creatine-free diet. Wild-type controls were included for all dietary manipulations.

Since AGAT^-/-^ mice are unable to biosynthesize creatine, the myocardial creatine levels are dependent on dietary intake. Two types of study were performed based on the following diets and summarized in *Figure 1*: (i) Creatine withdrawal study: mice were bred and weaned onto a chow supplemented with creatine monohydrate (0.5%, w/w), then switched to a standard creatine-free chow at 4–5 months. (ii) Creatine-naïve mice and dietary rescue: AGAT^-/-^ mice fed a creatine-free chow throughout development are termed ‘creatine-naïve’ and have whole-body creatine deficiency. Dietary creatine was introduced in adulthood by switching to a standard diet with 0.5% (w/w) creatine for either 1 week or 7 weeks. l-Homoarginine hydrochloride (HA, Sigma–Aldrich, UK) was added to the drinking water at a concentration of 14 mg/L for 10 days.^18^^,^^23^

### 2.3 *In vivo* cardiac phenotyping

All *in vivo* magnetic resonance experiments were carried out under isoflurane anaesthesia on a 9.4 T (400 MHz) MR system (Agilent Technologies) and using a quadrature-driven birdcage resonator (Rapid Biomedical) for high-resolution Magnetic Resonance cine Imaging (cine MRI) and cardiac ^1^H-MRS. Haemodynamic measurements in the left ventricle were made under isoflurane anaesthesia using a 1F-miro-tip catheter (Millar, Texas, USA) as previously described.^12^ Dobutamine was infused at 16 ng/g BW/min via the jugular vein as a measure of contractile reserve. Mice were killed by cervical dislocation at the end of the experiments and their organs removed, washed in heparinised saline, blotted, and weighed.

### 2.4 Isolated perfused heart function

Creatine naïve AGAT^-/-^ (*n* = 8) and WT (*n* = 7) mice were anaesthetized with sodium pentobarbital, hearts rapidly excised, cannulated and perfused in Langendorff constant pressure mode at 80 mmHg with oxygenated Krebs–Henseleit buffer at 37 °C. LV function was assessed in spontaneously beating hearts using a water-filled intraventricular balloon with end-diastolic pressure set to 6.1 ± 0.7 mmHg.

### 2.5 Isolated cardiomyocyte function

LV cardiomyocytes were isolated via enzymatic dispersion from WT and AGAT^-/-^ mice that had been fed a creatine containing diet throughout life. Cell shortening and re-lengthening velocity were measured under field-stimulation (3 Hz; 35 ± 1 °C) using a video-edge detection system (IonOptix Corp). In a sub-set of cardiomyocytes, the [Ca^2+^]_*i*_ transient was measured in fura-2 loaded (5 µmol/L, Molecular Probes) myocytes.

### 2.6 Biochemistry

High-energy phosphates were measured in isolated perfused hearts by ^31^P-MRS as previously described.^11^ Plasma HA levels were quantified in mouse plasma using a stable isotope dilution assay for LC–MS/MS.^24^ Creatine and taurine levels were quantified by solution-state ^1^H-NMR spectroscopy in LV tissue (∼50 mg) from creatine-naïve mice (three AGAT^-/-^ and three WT, age-matched females, mean 30 weeks). Total citrate synthase, adenylate kinase (AK) activity, CK activity, and CK isoenzyme composition were measured in LV homogenates.

### 2.7 Mitochondrial respiration

Cardiac mitochondria were isolated from creatine-naïve AGAT^-/-^ (*n* = 8) and WT (*n* = 7) and basal respiration assessed with a Clark-type electrode using the Mitocell S200A Micro Respiratory System (Strathkelvin Instruments, Motherwell, UK) using glutamate (5 mM), malate (2.5 mM), and Na^+^-pyruvate (5 mM) as substrates.

### 2.8 Cardiomyocyte cross-sectional area

Hearts from creatine-naïve mice were fixed in 10% buffered formalin, dehydrated, and embedded in paraffin. Eight-micron-thick sections were stained with Masson’s trichrome and measurements were made on ∼150–200 myocytes per animal.

### 2.9 Body composition

Body composition was measured in conscious mice using an EchoMRI-100 quantitative magnetic resonance whole body composition analyzer (Echo Medical Systems, Houston, USA).

### 2.10 Data analysis

Analysis of haemodynamics, MR data, single cell experiments, and all biochemistry measurements were performed independently and analysed in a blinded fashion. Data are expressed as mean ± SD unless otherwise stated. The following statistical tests were used: paired two-tailed *t*-test for comparison of AGAT^-/-^ before and after creatine withdrawal, unpaired two-tailed *t*-test for comparison of AGAT^-/-^ vs. WT, Mann–Whitney *U* test for comparison of AGAT^-/-^ vs. WT in cases where normal distribution was violated, 1-way ANOVA for multiple group comparisons, and 2-way ANOVA to assess the effect of genotype and treatment on body composition and for comparison of the haemodynamic response to dobutamine. For all single cell variables, analysis was carried out in RStudio using a hierarchical statistical method.^25^ In cases where variables was non-normally distributed, data were logarithmically transformed prior to statistical analysis. Bonferroni’s correction for multiple comparisons was used throughout this study with a significance level of *P* < 0.05.

## 3. Results

**A. Creatine withdrawal study:** Since AGAT^-/-^ mice are dependent on dietary creatine, we hypothesized that changing to a creatine-free diet in adulthood would lead to a gradual reduction in myocardial creatine levels to demonstrate whether low creatine, as observed in the failing heart, can cause cardiac dysfunction *per se*. *Figure 1A* shows a schematic of the experimental protocol.

### 3.1 Dietary creatine withdrawal leads to myocardial creatine loss

**Figure 2:**
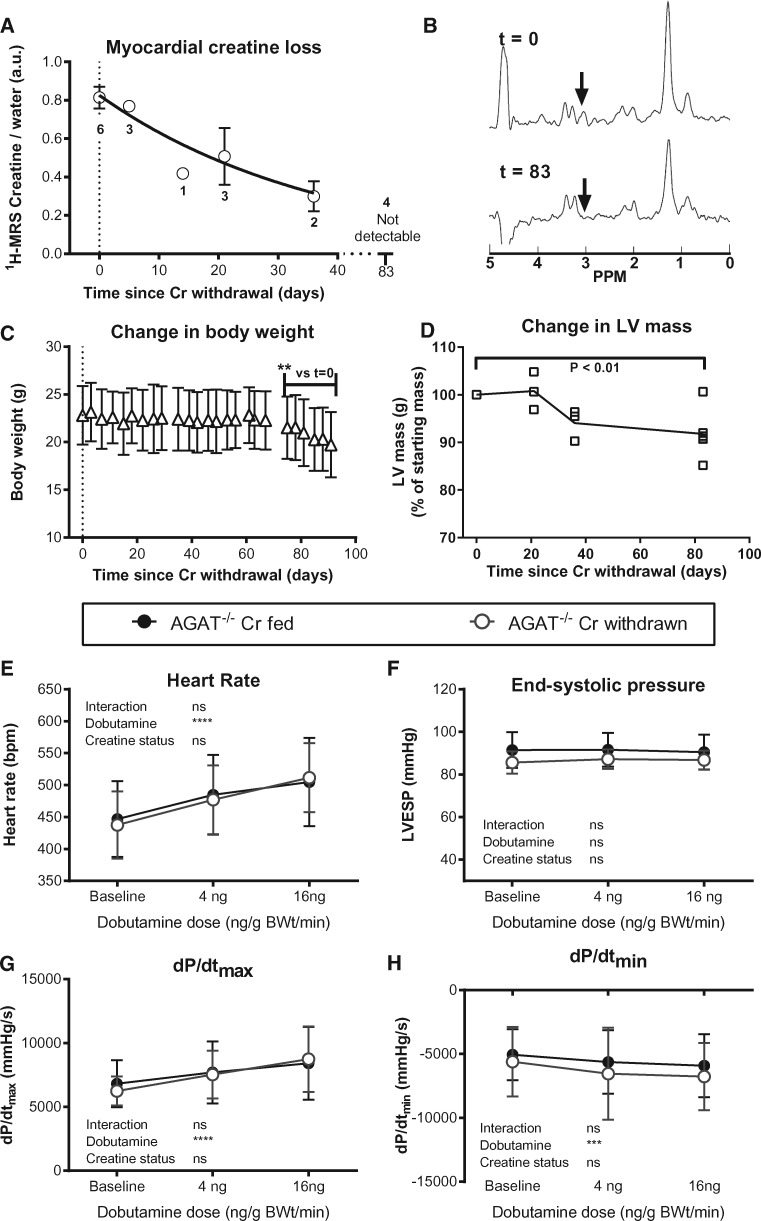
Withdrawal of dietary creatine in AGAT^-/-^ mice reduces body weight and LV mass without affecting haemodynamic function. (*A*) Myocardial creatine depletion was estimated at 2.7 ± 0.4% of the free creatine pool per day using *in vivo*^1^H-MRS. Data were fitted using a kinetic model of non-enzymatic degradation, according to the following equation: [Cr]_*t*_ = [Cr]_*t*__=0_. e^-^^*kt*^. (*B*) Representative ^1^H-MRS spectra of the same mouse before and after creatine withdrawal. The creatine peak (arrow) seen at day 0 is not visible by day 83. (*C*) Body weight decreased rapidly after 70 days of creatine-free diet (*n* = 6). (*D*) LV mass calculated by *in vivo* cine-MRI falls during dietary creatine withdrawal. LV haemodynamic parameters were measured at day 90 in AGAT^-/-^ mice with and without dietary creatine withdrawal (*n* = 6/group) under resting baseline conditions and with IV infusion of dobutamine. There were no significant differences between groups for (*E*) heart rate, (*F*) LV end-systolic pressure, (*G*) the rate of pressure rise maximum (dP/dt_max_) as a measure of contractility, or (*H*) the rate of pressure rise minimum (dP/dt_min_) as a measure of relaxation. Comparison was made by two-way repeated measures ANOVA and data are represented as mean ± SD.

Myocardial creatine levels were measured non-invasively using ^1^H-MRS before and after dietary creatine withdrawal in six adult AGAT^-/-^ mice. At day 0, myocardial creatine levels were similar to those of WT at 75 ± 10 nmol/mg protein (cf. 68 ± 5 for WT in *Table 1*) and were undetectable using ^1^H-MRS by day 83 (*Figure 2A* and *B*). Creatine loss followed a clear exponential decay pattern with a calculated constant rate of 2.7 ± 0.4% of the total creatine pool lost per day. However, residual creatine was detected by HPLC at day 91 (22.8 ± 2.5 nmol/mg protein), suggesting that part of the creatine pool may not have been visible on MR *in vivo*.

**Table 1:** Metabolites and markers of hypertrophy in WT and AGAT^-/-^ creatine-naïve hearts

	Wild-type	AGAT^-/-^ Cr-naïve
*^31^P-MRS*	(*n* *=* *6*)	(*n* *=* *4)*
PCr (mM)	12.7 ± 2.3	0
ATP (mM)	7.0 ± 0.4	6.9 ± 0.8
Pi (mM)	1.5 ± 0.5	4.1 ± 1.8*
pHi	6.9 ± 0.1	7.0 ± 0.1
*HPLC*	(*n* *=* *5*)	(*n* *=* *5*)
Total creatine (nmol/mg protein)	68 ± 5	<12 ± 1
Total adenine nucleotides (nmol/mg protein)	38 ± 11	42 ± 3
*Solution state ^1^H-NMR*	(*n* *=* *3*)	(*n* *=* *3*)
Creatine (normalized intensity)	92.0 ± 1.1	1.5 ± 0.6****
Taurine (normalized intensity)	0.17 ± 0.02	0.21 ± 0.01*
*Post-mortem weights*	(5M/5F)	(5M/5F)
Body weight (g)	28.1 ± 6.1	13.4 ± 2.0***
Tibia length (mm)	17.9 ± 0.6	17.1 ± 0.3**
LV weight (mg)	83 ± 14	61 ± 4***
LV/BWt (mg/g)	2.98 ± 0.28	4.62 ± 0.49***
LV/tibial length (mg/mm)	4.59 ± 0.71	3.56 ± 0.23***
RV weight (mg)	23.5 ± 4.6	15.1 ± 1.7***
*Hypertrophic markers*	(*n* *=* *9*)	(*n* *=* *10*)
ANP mRNA (% WT)	100 ± 42	420 ± 198***
BNP mRNA (% WT)	100 ± 28	134 ± 87
β-MHC mRNA (% WT)	100 ± 28	260 ± 335
α-SA mRNA (% WT)	100 ± 57	36 ± 19**
*Histology*	(*n* *=* *3*)	(*n* *=* *3*)
Myocyte cross-sectional area (µm^2^)	198 ± 11	210 ± 70

LV, left ventricle; RV, right ventricle, ANP, atrial natriuretic peptide; BNP, brain natriuretic peptide; β-MHC, beta myosin heavy chain; α-SA, alpha skeletal actin. Values are represented as mean ± SD.

* *P* < 0.05.

** *P* < 0.01.

*** *P* < 0.001.

**** *P* < 0.0001 vs. WT.

### 3.2 Creatine withdrawal alters body weight but not cardiac function

Body weight was stable until day 60 of creatine withdrawal, after which we observed gradual and persistent weight loss of ∼0.5% of the initial body weight per day. After 91 days on a creatine-free diet, mice had lost 14% body weight (*P* < 0.001), which required the termination of the experiment for animal welfare reasons (*Figure 2C*). Cine-MRI examination during the withdrawal period showed a commensurate reduction in LV mass, which was 8% lower by the final time point (*P* < 0.01; *Figure 2D*). During this period, there was no change in cardiac function assessed non-invasively using cine-MRI, that is, ejection fraction and cardiac output remained stable (see Supplementary material online, *Table S1*).

*In vivo* LV haemodynamic measurements were made at the final time point before and after maximal β-adrenergic stimulation with dobutamine (*Figure 2E*–*H*). There were no significant differences between AGAT^-/-^ mice following creatine withdrawal and the control group of AGAT^-/-^ mice fed creatine throughout the experiments. For example, LV pressures, parameters of contraction (dP/dt_max_), and relaxation (dP/dt_min_) were all indistinguishable from Cr-fed AGAT^-/-^ controls. Moreover, contractile reserve, the increase in function observed upon dobutamine stimulation, which is a sensitive marker for early cardiac dysfunction, was unaltered. Post-mortem data confirmed reduced LV mass and lung weights in mice withdrawn from Cr compared with the control group (see Supplementary material online, *Table S1*).

**B. Creatine-naïve mice and dietary rescue:** Since cardiac function was unaffected by dietary creatine withdrawal, we sought to study the extreme scenario of absolute creatine-deficiency, to determine whether complete absence of creatine causes cardiac dysfunction. For these experiments we used AGAT^-/-^ mice from a colony that had always been maintained on a creatine-free diet (i.e. creatine-naïve). To determine causality for the observed phenotypes we performed dietary rescue experiments as per the schematic in *Figure 1B*.

### 3.3 Creatine-naïve AGAT^-/-^ hearts are devoid of creatine

**Figure 3:**
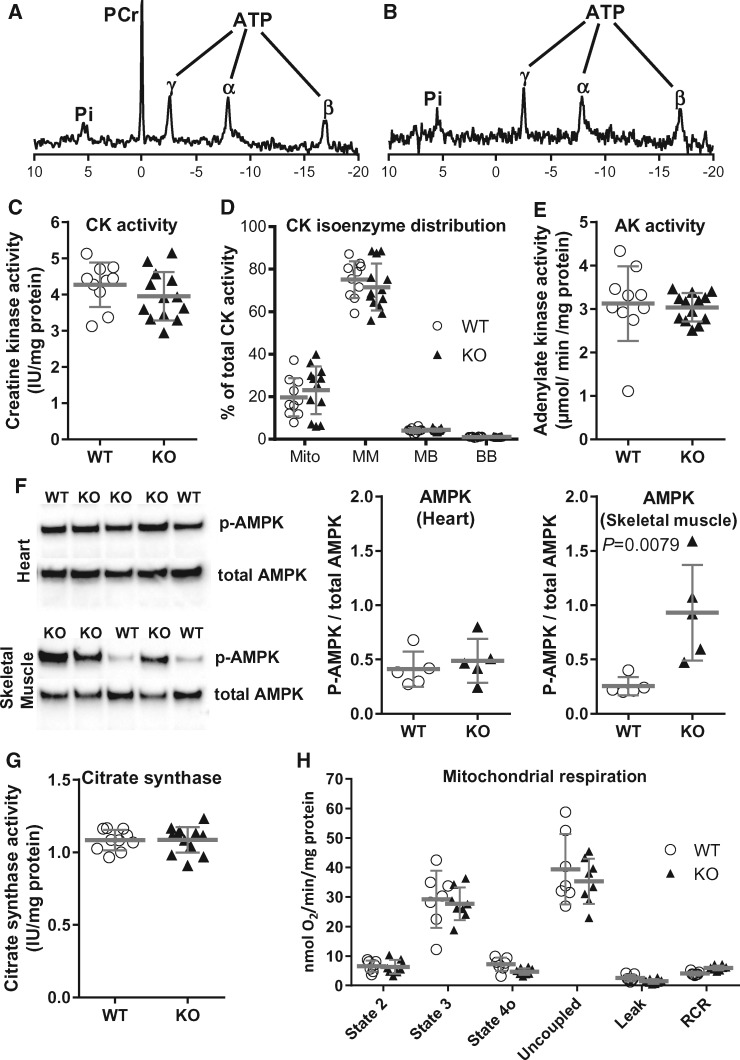
Absence of phosphocreatine (PCr) in creatine-naïve AGAT knockout mice does not alter key metabolic parameters in the heart. Representative ^31^P-magnetic resonance spectra in Langendorff-perfused hearts. PCr was the most prominent peak in hearts from wild-type mice (*A*), but was completely absent in hearts from creatine-naïve AGAT^-/-^ mice (*B*), where inorganic phosphate (Pi) was elevated, and there was no change in ATP (appears as three peaks representing the γ, α, and β phosphoryl groups). Key energy homeostasis enzymes and mitochondrial function were not significantly different between wild-type (WT) and creatine-naïve AGAT^-/-^ (KO) hearts. (*C*) Total creatine kinase (CK) activity and percentage isoenzyme distribution (*D*), where Mito is mitochondrial CK and the various dimers of Muscle and Brain isoforms; (*E*) adenylate kinase (AK) activity (all *n* = 10 WT, *n* = 13 KO). (*F*) AMPK activation expressed as the ratio of phospho- to total AMPK protein expression was not altered in LV, but was significantly elevated in hind-limb skeletal muscle, *n* = 5 per group. (*G*) Citrate synthase activity (*n* = 10 WT, *n* = 13 KO) and (*H*) mitochondrial respiration with glutamate (5 mM), malate (2.5 mM) and Na^+^-pyruvate (5 mM) as substrates (*n* = 7 WT, *n* = 8 KO). Values are mean ± SD.

Hearts were perfused in Langendorff mode for assessment of high-energy phosphates by ^31^P-MRS. The complete absence of PCr in AGAT^-/-^ hearts (*Figure 3A* and *B*) was most striking and resulted in elevated inorganic phosphate, while ATP and intracellular pH remained at normal wild-type (WT) levels (*Table 1*). Total adenine nucleotides (i.e. AMP + ADP + ATP) were unchanged. Determination of total creatine by HPLC could not resolve a peak above background noise, and we thus confirmed, using solution state ^1^H-NMR, that creatine levels were negligible. The cellular osmolyte taurine was elevated by 24% in AGAT^-/-^ hearts, which may partially compensate for the osmotic stress caused by creatine deficiency.

### 3.4 Creatine-naïve hearts do not exhibit cardiac hypertrophy

Whole-body creatine deficiency resulted in severely reduced body weight (52% lower), but with a disproportionate reduction in long bone length (tibial length 4% shorter), which complicated the interpretation of organ weights. For example, absolute LV weight was significantly lower in age-matched AGAT^-/-^ mice compared with WT, confirmed when normalized to tibial length, but is significantly higher than WT when normalized to body weight (*Table 1*). We therefore determined the gene expression of molecular markers of hypertrophy (i.e. ANP, BNP, β-MHC, α-SA), which were not consistently elevated in AGAT^-/-^ hearts (one marker was elevated, another one was reduced, and two other markers were unchanged). Absence of a molecular programme of hypertrophy was confirmed at the cellular level by histology, with the myocyte cross-sectional area found to be unaltered (*Table 1*).

### 3.5 Creatine-naïve hearts and markers of energy homeostasis

We analysed the expression of key energy homeostasis enzymes for evidence of compensatory adaptation. The total absence of substrate resulted in a 2.1-fold up-regulation of creatine transporter gene expression (*P* = 0.02), but the activity of CK and the distribution of CK isoenzymes were unaltered (*Figure 3C* and *D*). AK may also play a phospho-transfer role in the heart and can compensate for the loss of CK system function; however, AK activity was also unaltered in creatine-naïve AGAT^-/-^ hearts (*Figure 3E*). Phosphorylation of AMP-activated protein kinase (AMPK) is a common indicator of impaired energetic status, but AMPK was not activated in the AGAT^-/-^ heart (*Figure 3F*). This was surprising since previous studies showed AMPK activation in skeletal muscle from AGAT^-/-^ mice,^22^ and we therefore sought to confirm this as a positive control for our own assay. AMPK was indeed activated in skeletal muscle of our AGAT^-/-^ mice (*Figure 3F*), indicating a divergence of the biochemical consequences of creatine depletion in cardiac as compared with skeletal myocytes. AMPK activation in AGAT^-/-^ skeletal muscle stimulates the PGC1-α mitochondrial biogenesis pathway and thereby increases citrate synthase activity (a marker for mitochondrial volume).^15^ In agreement with the lack of AMPK activation in heart, we observed no change in citrate synthase activity in cardiac muscle of AGAT^-/-^ (*Figure 3G*). Furthermore, there was no difference in baseline or ADP-stimulated respiration in mitochondria isolated from WT and AGAT^-/-^ hearts (*Figure 3H*).

### 3.6 Creatine-naïve mice have altered body composition

**Figure 4:**
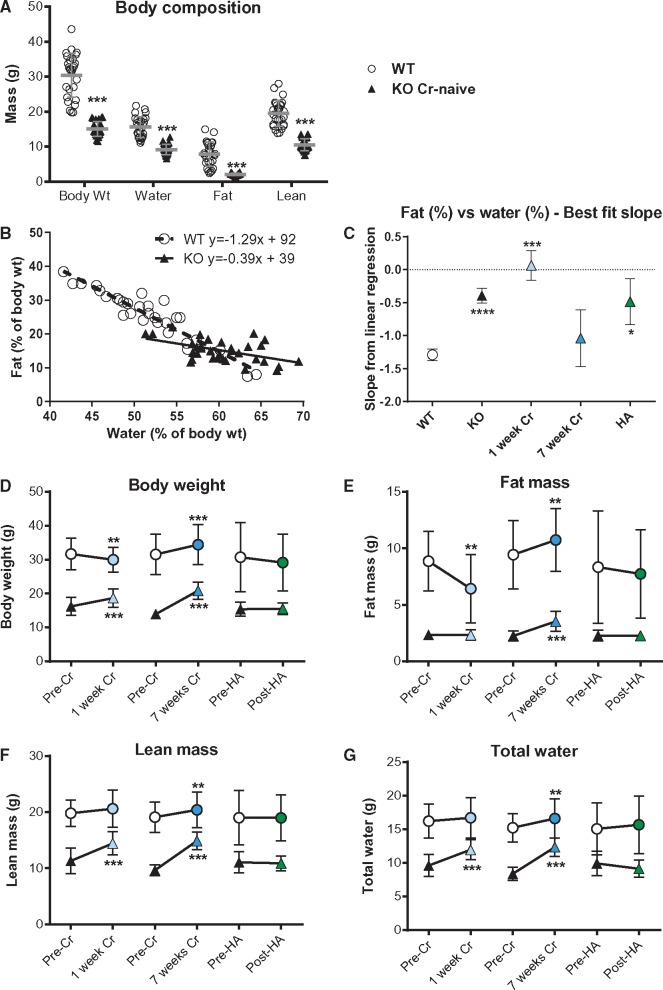
Creatine-naïve AGAT^-/-^ mice have low body weight and altered composition rescuable by dietary creatine. (*A*) Creatine-naïve KO mice had low body weight associated with reduced water, fat and lean mass (***P < 0.001; *n* = 14 male and 14 female per group). (*B*) These changes were not proportional because the linear relationship between % body fat and % total water was significantly altered in KO mice (*P* < 0.0001 for slope). Dietary supplementation with 0.5% creatine for 1 week abolished the fat-water relationship, which was rescued to WT values after 7 weeks of dietary creatine and was unaltered by homoarginine (HA) supplementation (*C*). Values for body weight (*D*), fat mass (*E*), lean mass (*F*), and total water (*G*) in the same mice before and after 1-week and 7-week creatine supplementation or 10-day homoarginine supplementation are shown in WT (open circles) and KO (triangles). Lean mass and total water were rapidly changing, suggesting an osmotic role for creatine, whereas fat mass only changed with chronic dietary creatine. Each data point represents mean ± SD for *n* = 7–10 mice except HA wild-type (*n* = 4), ** denotes *P* < 0.01, *** *P* < 0.001, **** *P* < 0.0001 compared with pre-treatment values.

**Figure 5:**
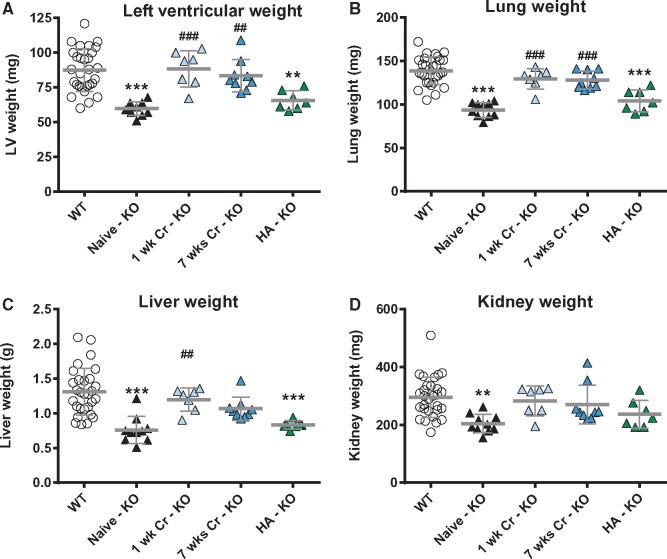
Creatine-naïve AGAT^-/-^ mice have low vital organ weights that are rapidly rescued by dietary creatine supplementation. Post-mortem blotted organ weights from left ventricle (*A*), lung (*B*), liver (*C*), and kidney (*D*) taken from wild-type (WT, *n* = 29), creatine-naïve knockout (KO, *n* = 10), and KO mice supplemented with 0.5% dietary creatine for 1 week (*n* = 7), 7 weeks (*n* = 9), or homoarginine (HA) 14 mg/L added to the drinking water (*n* = 7). Data are represented as mean ± SD, ** denotes *P* < 0.01 and ** *P* < 0.001 compared with WT and ^##^*P* < 0.01, ^###^*P* < 0.001 compared with creatine-naïve knockout.

Body composition measured non-invasively by MR relaxometry showed that AGAT^-/-^ mice had significantly lower lean mass and markedly reduced body fat and water content (*Figure 4A*). The relationship between percentage fat and water was highly linear over a wide range of body weights,^26^ as can be seen for WT mice in *Figure 4B*. For AGAT^-/-^, the slope was significantly altered (*P* < 0.0001), suggesting a fundamental breakdown in this relationship. To establish the role of creatine on body composition, we supplemented the diet of AGAT^-/-^ mice with 0.5% creatine monohydrate for 1 week, which is sufficient to normalize tissue levels (e.g. in myocardium [Cr] WT 74 ± 3 vs. AGAT^-/-^ 70 ± 2 nmol/mg protein). A 7-week creatine supplementation was also included to determine the long-term consequences. A further group consisted of AGAT^-/-^ mice supplemented with 14 mg/L HA added to the drinking water for 10 days in order to rule out a role for HA deficiency. After 1 week of creatine feeding, the linear relationship between body fat and water was abolished (slope = 0), whereas the relationship was indistinguishable from WT by 7 weeks and unaffected by HA (*Figure 4C*). Body composition analysis before and after supplementation showed a rapid increase in lean mass and body water by 1 week (*Figure 4F*–*G*), which is consistent with creatine having both an ergogenic and osmotic role. A gain in fat mass was not observed until after 7 weeks (*Figure 4E*), which restored the fat-water relationship (*Figure 4C*). These observations are likely to provide an explanation for the low post-mortem organ weights in AGAT^-/-^ mice, which were rescued within one week of creatine supplementation (*Figure 5*), suggesting that creatine acts as a compatible osmolyte in the heart and other major organs (i.e. low LV weight is due to reduced water content).

### 3.7 Creatine-naïve mice have smaller LV chamber volumes

WT and creatine-naïve AGAT^-/-^ mice underwent cine-MRI to assess global structure and function *in vivo* (see Supplementary material online, Figure). The difference in LV size is clearly evident in the representative mid-ventricular short-axis images and is reflected in the low LV mass and small ventricular end-diastolic and end-systolic volumes in creatine-naïve AGAT^-/-^ mice. We observed a significant reduction in cardiac output driven by trends in both heart rate and stroke volume, but with preserved ejection fraction. The physiological significance is open to interpretation given the large differences in body weight and composition. We therefore performed LV haemodynamic measurements, which are independent of chamber size.

### 3.8 Creatine-naïve hearts exhibit haemodynamic impairment

**Figure 6:**
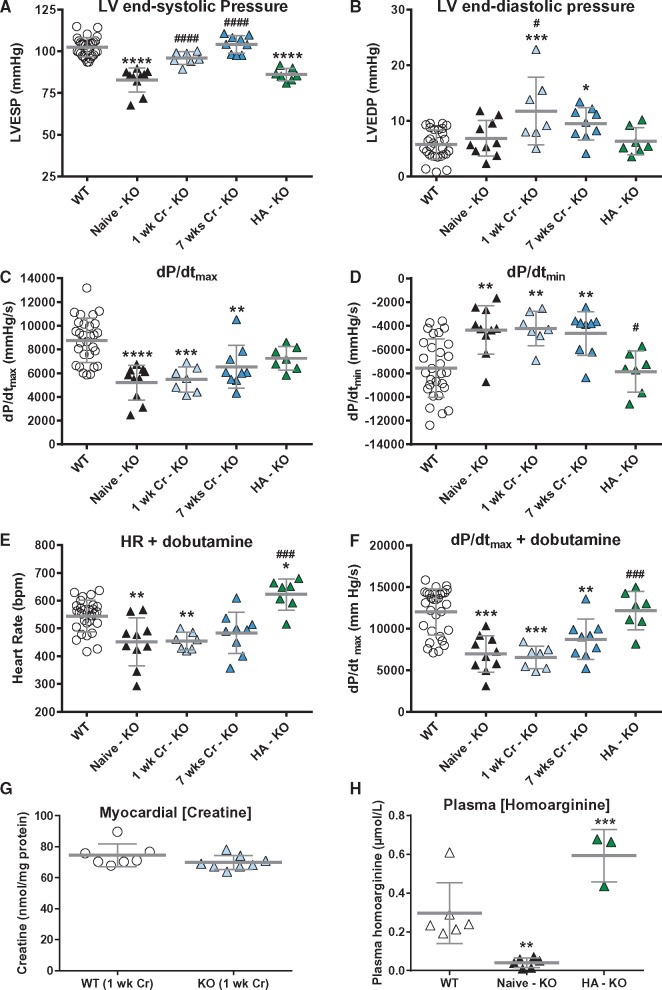
*In vivo* haemodynamic measurements in creatine-naïve AGAT^-/-^ (KO) mice shows inotropic and lusitropic deficits rescued by homoarginine but not by creatine supplementation. (*A*) LV end-systolic pressure, (*B*) LV end-diastolic pressure, (*C*) the rate of pressure rise maximum (dP/dt_max_) as a measure of contractility, (*D*) the rate of pressure rise minimum (dP/dt_min_) as a measure of relaxation. (*E*) and (*F*) are heart rate and dP/dt_max_, respectively during IV infusion with dobutamine at 16 ng/g BW/min. WT control and treatment groups did not significantly differ for any of the parameters and were subsumed into one group (*n* = 29), all other groups *n* = 7–10. (*G*) Supplementation with 0.5% dietary creatine for 1 week normalized myocardial creatine levels (*n* = 7–8). (*H*) Plasma levels of homoarginine were significantly lower in KO (*n* = 6) vs. WT (*n* = 6) and are elevated by supplementation via drinking water (*n* = 3). All data are represented as mean ± SD, * denotes *P* < 0.05, ** *P* < 0.01, *** *P* < 0.001 and **** *P* < 0.0001 compared with WT and ^#^*P* < 0.05, ^##^*P* < 0.01, ^###^*P* < 0.001, ^####^*P* < 0.0001 compared with creatine-naïve knockout.

Compared with WT controls, creatine-naïve AGAT^-/-^ mice had a distinct haemodynamic phenotype consisting of lower LV systolic pressure with normal end-diastolic pressures and significantly impaired contractility and relaxation as shown by the reduced rates of pressure rise (dP/dt_max_) and fall (dP/dt_min_; *Figure 6A*–*D*). Maximal heart rate and dP/dt_max_ in response to dobutamine infusion was also lower in AGAT^-/-^ hearts, indicating an impaired contractile reserve (*Figure 6E* and *F*).

**(i) Creatine rescue:** In order to establish causality, we treated mice with 0.5% dietary creatine, either for 1 week or 7 weeks, to determine whether this would rescue the *in vivo* phenotype. WT control mice were included for each treatment group, but did not differ in any haemodynamic parameter and were therefore combined into a single WT control group (see Supplementary material online, *Table S2*).

In AGAT^-/-^ mice, creatine supplementation corrected myocardial creatine levels within one week (*Figure 6G*), normalized LV end-systolic pressure, and increased end-diastolic pressure (*Figure 6A and B*). This may reflect the osmotic effect of acute creatine replacement to increase muscle tone, because there was no apparent effect on functional parameters. For example, correcting creatine levels had no effect on either the inotropic or lusitropic deficits, or on contractile reserve.

**(ii) HA rescue:** Since creatine supplementation of AGAT^-/-^ mice did not fully rescue the cardiac phenotype and AGAT^-/-^ mice were also characterized by low HA plasma levels, we included a further group where we supplemented with HA via drinking water. Plasma HA levels were low in AGAT^-/-^ mice and were significantly elevated after 10 days of oral supplementation (*Figure 6H*). Surprisingly, all inotropic and lusitropic parameters (i.e. dP/dt_max_, dP/dt_min_ and response to dobutamine) were rescued by HA supplementation (*Figure 6C*–*F*).

**Figure 7:**
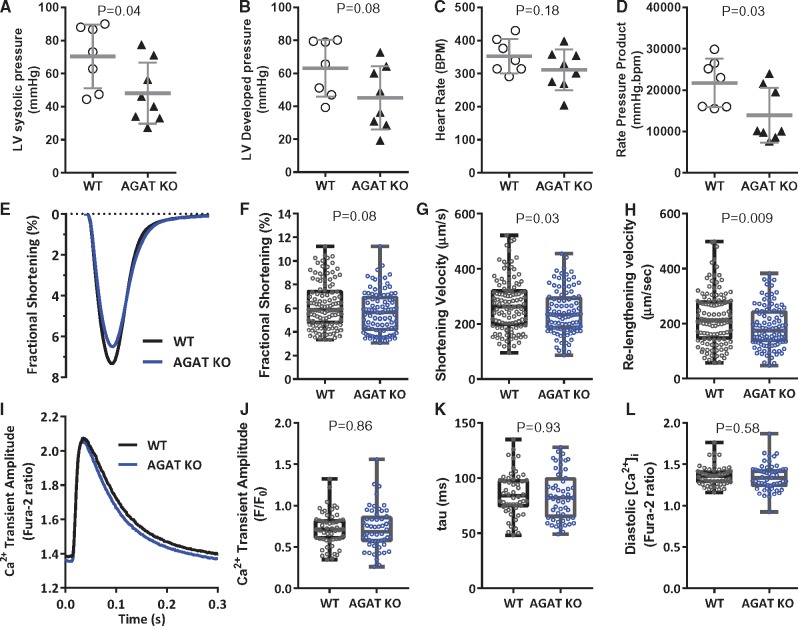
Contractile dysfunction is confirmed *ex vivo* in creatine-naïve hearts and a role for homoarginine-deficiency is confirmed in creatine-replete isolated cardiomyocytes. Hearts perfused in Langendorff mode from wild-type (WT; *n* = 7) and creatine naïve AGAT^-/-^ mice (KO; *n* = 8) showing (*A*) Left ventricular end-systolic pressure, (*B*) LV developed pressure, (*C*) Heart rate, (*D*) Rate pressure product. Mean values ± SD with * denoting *P* < 0.05 by two-way unpaired *t*-test. Cardiomyocytes were isolated from WT and AGAT^-/-^ mice supplemented with 0.5% dietary creatine (i.e. homoarginine deficiency only). (*E*) Averaged cell shortening recording in field-stimulated (3 Hz, 35 °C) LV myocytes. (*F*) AGAT^-/-^ cardiomyocytes show a trend for impaired fractional shortening and (*G*, *H*) slower shortening and re-lengthening kinetics compared with WT cardiomyocytes (*n* = 106/97 cells from seven hearts per genotype). This occurred in the absence of differences in [Ca^2+^]_*i*_ transient amplitude (*I*, *J*) the decay constant of the [Ca^2+^]_*i*_ transient (tau) (*K*) or in diastolic Ca^2+^ levels (*L*) (*n* = 51/53 cells from 6/6 hearts per genotype). Data are represented as median (IQR), *P* values were calculated by hierarchical statistical analysis on normally distributed data or on logarithmic transformed data (as indicated in Supplementary material online, *Table S3*).

**(iii) Isolated perfused heart:** The presence of cardiac dysfunction in creatine-naïve AGAT^-/-^ mice was confirmed *ex vivo* in Langendorff-perfused hearts (*Figure 7A*–*D*), suggesting that dysfunction is an intrinsic property and not secondary to altered loading conditions or differences in whole-body composition or metabolism.

### 3.9 HA deficiency impairs cardiomyocyte function

We sought to confirm the *in vivo* and *ex vivo* findings at the single cardiomyocyte level. For this we used WT and AGAT^-/-^ mice that had been fed a creatine-supplemented diet throughout life, i.e. a pure HA-deficiency without the potentially confounding effects of low creatine on cellular osmolarity, haemodynamic loading, and whole body metabolism. HA-deficient cardiomyocytes showed a modest reduction in fractional shortening with significantly reduced shortening and re-lengthening velocities (*Figure 7E*–*H*), suggesting that low HA levels *per se* can impair cardiomyocyte function. This was not associated with changes in [Ca^2+^]_*i*_ transient amplitude, the time constant of [Ca^2+^]_*i*_ transient decay (tau), or intracellular diastolic calcium levels (*Figure 7I*–*L*). See Supplementary material online, *Table S3* for details of statistical analysis.

## 4. Discussion

We used AGAT^-/-^ mice to study, for the first time, the cardiac consequences of absolute creatine deficiency, i.e. in the absence of other compensatory phosphagens. Our results offer multiple novel insights into myocardial creatine efflux, creatine as a cellular osmolyte, biochemical divergence between cardiac and skeletal muscle creatine, cardiac HA and their impact on cardiac function.

### 4.1 Myocardial creatine loss

Withdrawal of dietary creatine in AGAT^-/-^ mice allowed us to measure the rate of myocardial creatine efflux for the first time. Degradation of creatine and PCr to creatinine is a spontaneous, non-enzymatic and irreversible process. Creatinine is formed at a constant rate, diffusing into the blood before being filtered and excreted by the kidneys.^2^ Starting from WT levels, a decrease of ∼2.7% per day in myocardial creatine content was calculated, which is in close agreement with earlier estimates of whole-body creatine loss of ∼2% per day.^27^ Our experiments had to be terminated after three months of withdrawal due to excessive body weight loss, which triggered our humane end-point to prevent unnecessary animal suffering. Residual creatine was therefore present at the point of phenotyping and previous β-GPA experiments suggest that cardiac function is only impacted when less than ∼20–25% creatine remains.^10^^,^^28^ Nevertheless, final creatine levels were well below that observed in the failing heart without impacting on *in vivo* function or contractile reserve. This argues against a causative role for low creatine in driving contractile dysfunction. It should be noted that the effect of HA deficiency was not apparent in this experiment because we compared knockout groups with and without dietary creatine (i.e. both groups were HA deficient).

### 4.2 Creatine as compatible osmolyte

Weight loss or low body weight has been a universal finding in creatine deficiency studies, either due to β-GPA feeding or GAMT^-/-^, and creatine-naïve AGAT^-/-^ mice are even more severely affected.^22^ Our study was performed using non-invasive MR relaxometry, which allowed us to examine the effects on body composition before and after creatine rescue in the same mice. Our results demonstrated that fat mass, lean mass, and total water were lower in AGAT^-/-^ mice and was rescued by creatine supplementation. The robust increase in total water was most notable, occurring within one week of creatine supplementation, which strongly suggests that creatine acts as an osmolyte in the heart and other organs, such that replacing cellular creatine also replaces water. In line with this observation, we found elevated levels of the established osmolyte, taurine, in AGAT^-/-^ hearts, which could represent a partial compensation for the loss of creatine as an osmolyte. The converse was observed in mice with elevated myocardial creatine, in which taurine levels were negatively correlated with creatine, suggesting a degree of interchangeability, i.e. compatible osmolytes.^29^ This is supported by cell culture studies in which creatine was as effective as taurine in protecting cultured muscle cells following exposure to hypertonic media.^30^ Indeed, an osmotic effect of creatine is the most likely explanation for the complete normalization of organ weights within one week of creatine supplementation.

### 4.3 Cardiac adaptations to low creatine levels

Creatine-naïve mice were bred using heterozygous mating to restrict the effects on development. This implies that they may have received small amounts of creatine via the placenta and suckling, but our experiments were performed in adult mice aged >20 weeks in which the absence of creatine was verified by multiple methods. Post-natal compensatory adaptations have been described even for a relatively short β-GPA feeding period, among them mitochondrial proliferation^31^ and changes in myosin isoenzyme expression associated with ventricular hypertrophy,^6^ although these may represent off-target effects of β-GPA. We cannot completely rule out the development of adaptations in AGAT^-/-^ hearts, although we did not observe changes in mitochondrial respiration or citrate synthase activity (a marker of mitochondrial volume), nor did we observe cardiac hypertrophy at a histological or molecular level. In CK-deficient mice, mitochondrial rearrangement was described to reduce diffusion distances for high-energy phosphates,^32^ but detailed analysis of GAMT^-/-^ hearts failed to detect similar alterations of creatine deficiency.^33^

Other obvious adaptations were absent, e.g. AK represents an alternative phosphotransfer mechanism that is upregulated in CK-deficient mouse hearts.^34^ We did not observe changes in AK activity in AGAT^-/-^ mice, although a definitive answer would require measurements of AK flux. These findings are in agreement with the GAMT^-/-^ model, where differential proteomic analysis did not reveal any potential adaptations.^12^ Most importantly, we can rule out the contribution of GA participation to the CK reaction as a compensatory mechanism for the GAMT^-/-^ cardiac phenotype.

### 4.4 Skeletal vs. cardiac muscle phenotype

In contrast to the heart, AGAT^-/-^ mice have severe muscular dystrophy manifesting as skeletal muscle atrophy, abnormal mitochondria, and reduced grip strength, all of which were completely rescued by creatine supplementation.^15^ The fundamental difference appears to be that ATP is maintained at normal levels in the heart, but was 46% lower in skeletal muscle.^15^ Unsurprisingly, this energetic deficit leads to the activation of AMPK, which we confirmed in skeletal muscle, but which was absent in cardiac muscle within the same animals. AMPK is an important energy sensor acting to switch off energy-demanding processes and activating energy-saving and production-related pathways,^35^ as demonstrated by multiple changes in metabolic gene expression observed in AGAT^-/-^ skeletal muscle.^36^ One downstream consequence is the stimulation of mitochondrial biogenesis via the PGC1-α pathway, which resulted in an elevation of the citrate synthase activity by 70% in skeletal muscle,^15^ but we did not observe any changes in the heart. This demonstrates a major divergence in the biochemical consequences of creatine depletion in skeletal vs. cardiac muscle, perhaps reflecting higher mitochondrial cell density and capacity for oxidative phosphorylation in cardiomyocytes,^37^ making them less reliant on the CK system to maintain ATP. That PCr levels are considerably higher in skeletal muscle,^2^ supports the view of a relatively CK-dependent tissue, whereas the heart is too important to fail and therefore displays greater metabolic redundancy.

A comparison of the skeletal muscle phenotype with GAMT^-/-^, too, provides valuable insight. GAMT^-/-^ mice do not have overt muscle weakness and can run just as far and as fast as WT controls.^12^ GA, the creatine precursor, has been shown to accumulate in both muscle types, where it can be phosphorylated by the CK reaction.^11^^,^^13^ Apparently, this is sufficient to compensate for creatine deficiency in skeletal muscle, hence the severe phenotype when GA is absent in AGAT^-/-^. In contrast, both GAMT^-/-^ and AGAT^-/-^ hearts show impaired contractile reserve,^11^ but this reflects HA deficiency in AGAT^-/-^. As GAMT^-/-^ mice are not HA deficient,^18^ it may be possible that GA accumulation accounts for the limited contractile reserve in this model, e.g. by inhibition of the Na^+^/K^+^ pump.^14^

### 4.5 Myocardial creatine is unnecessary to support baseline cardiac function

We did not observe any baseline dysfunction in creatine-naïve AGAT^-/-^ mice by cine-MRI, and this is in broad agreement with the earlier analogue feeding studies in which baseline dysfunction was found to be either relatively mild (e.g.^5–7^) or completely absent (e.g.^8–10^), with dysfunction becoming apparent or exacerbated at higher workloads. The GAMT^-/-^ model, too, is in agreement with this general pattern, showing normal function by cine-MRI and only a small reduction in LV systolic pressure at baseline.^11^ Our findings in AGAT^-/-^ mice do not include potentially confounding effects of β-GPA or GA accumulation and support the concept that a fully functioning CK system is not required to maintain baseline cardiac function. Furthermore, we show that low creatine on its own is insufficient to drive cardiac dysfunction.

It is a limitation that our model is a global knockout, however this is unavoidable, since AGAT is predominately expressed in the kidney but not in the normal heart,^38^ so a cardiac-specific AGAT^-/-^ would be uninformative since creatine and HA are both taken up from the circulation. This means our *in vivo* haemodynamic phenotype could be confounded by changes in body composition, loading conditions and whole-body metabolism. For example, reduced dP/dt_max_ in creatine-naïve mice may have resulted from reduced load rather than from altered contractility. However, the fact that cardiac dysfunction in the creatine-naïve knockout persists in the *ex vivo* perfused heart, where loading conditions and metabolic substrates are controlled, strongly argues against whole-body confounders. Furthermore, we observed slower contraction and relaxation in isolated cardiomyocytes from HA-deficient (but creatine-replete) hearts. These experiments also eliminate differences in cellular osmolarity, and suggests that low HA levels *per se* may contribute to cardiac dysfunction. The magnitude of this effect is relatively modest and these changes were not explained by consonant changes in intracellular calcium. Our data do not rule out the potential for synergy when both HA and creatine levels are low.

### 4.6 HA and cardiac function

It is notable that low circulating HA has been identified as a novel risk factor for multiple cardio- and cerebrovascular diseases (reviewed in^21^). For example, in a prospective study of patients undergoing coronary angioplasty, low serum HA was independently associated with a higher risk of all-cause and cardiovascular mortality,^20^ including stroke, sudden cardiac death, fatal myocardial infarction, and heart failure, with a positive correlation between HA levels and ejection fraction.^39^ Collectively, these studies indicate that low plasma HA is a biomarker for cardiovascular disease risk, but the linking mechanism has yet to be identified. Notably, a causal relationship between HA deficiency and ischaemic stroke has been demonstrated in AGAT^-/-^ mice, which developed larger cerebral injuries that were rescued by HA supplementation.^18^

Our analogous findings provide the first evidence that low HA *per se* may contribute to impaired *in vivo* cardiac function, suggesting a potential role in the pathophysiology of heart disease. This is supported by our recent study demonstrating that HA supplementation in WT mice with ischaemic heart failure preserved contractile reserve.^23^

Our current study may also shed light on why AGAT expression is up-regulated in the human failing heart.^40^ Local creatine biosynthesis has been postulated, but seems unlikely in the absence of commensurate GAMT expression. An alternative explanation is that compensatory HA biosynthesis may support contractile function.

Finally, our findings may also be of relevance to patients with AGAT deficiency syndrome. This rare genetic disorder typically manifests in childhood as skeletal muscle myopathy and developmental delay, which responds to early creatine supplementation.^41^ Cardiac involvement has not been examined in these patients, but if confirmed to be present, our study predicts that HA supplementation would be beneficial.

### 4.7 Conclusions

Our findings represent the strongest evidence to date that a fully functioning CK system is not required for maintaining normal baseline cardiac function, or for supporting contractile reserve. Indeed, *in vivo* cardiac dysfunction in AGAT^-/-^ mice is principally driven by HA deficiency rather than creatine deficiency. This suggests that low HA is more than just a risk factor for cardiovascular disease, but may play an active role in its pathophysiology.

## Supplementary material

Supplementary material is available at *Cardiovascular Research* online.

## Supplementary Material

Supplementary Data
