# Supplementary material for: Impaired cardiac contractile function in arginine:glycine amidinotransferase knockout mice devoid of creatine is rescued by homoarginine but not creatine
Source: Cardiovasc Res. 2017 Dec 11;114(3):417–30. doi: 10.1093/cvr/cvx242 (PMC5982714; doi:10.1093/cvr/cvx242)
Supplement: Supplementary Data [file cvx242_supplementary_data_revision2.docx]

**SUPPLEMENTARY DATA**

**Detailed Methods**

**Animal generation and husbandry**

Arginine:glycine amidinotransferase knockout mice (AGAT^-/-^) have a homozygous knockout of the *Gatm^tm1.1Isb^* allele generated by homologous recombination in the laboratory of Prof. Dirk Isbrandt as previously described.[^1^](#_ENREF_1) They were imported to Oxford and have been backcrossed for >10 generations onto a C57BL/6J background. Mice for these experiments were bred by intercrossing heterozygous animals and genotyped by polymerase chain reaction. As soon as practicable (~4-6 weeks of age), mice were housed according to genotype in order to prevent ingestion of creatine via coprophagia. Mice were kept in specific pathogen-free cages and maintained on a 12/12 h light-dark cycle and under conditions of controlled temperature (20-22°C) and humidity. Water and chow were available ad libitum and all procedures were performed 4 to 6-month-old mice.

**Dietary supplementation**

The creatine status was defined based on the following diets: 1) Mice for creatine withdrawal studies were fed on a creatine-supplemented chow from weaning onward: R/M-H complete maintenance diet with addition of 5 g/kg creatine monohydrate (Ssniff, Soest, Germany) and were switched to Teklad Global 19% extruded rodent diet for creatine withdrawal (Harlan, UK). 2) Mice are referred to as *creatine-naïve* if they were fed Teklad Global 19% protein extruded rodent diet, which is naturally free of creatine, throughout the experiments. 3) Creatine rescue experiments were designed to supplement the Teklad diet with 0.5% w/w creatine monohydrate for either 1 week or 7 weeks. One week was chosen based on pilot data confirming the normalization of myocardial creatine levels, and the 7-week period was chosen to allow chronic adaptive changes to low-creatine levels to be reversed (this time-frame is sufficient to completely reverse the cardiac phenotype following removal of an aortic band.[^2^](#_ENREF_2)) L-homoarginine (Sigma-Aldrich, UK) was added to the drinking water at a concentration of 14mg/L for 10 days and given to creatine-naïve mice.

**Creatine withdrawal protocol**

In six AGAT^-/-^ mice (2M, 4F) myocardial creatine levels and cardiac function were assessed by ^1^H-MRS and cine-MRI before creatine was withdrawn from the diet and at multiple time-points thereafter. The rate of creatine loss was estimated using a kinetic model of non-enzymatic degradation, according to the following equation: [Cr]_t_= [Cr]_t=0_ . e^-kt^ (where [Cr]_t_ is the myocardial creatine level as estimated by ^1^H-MRS at the time t (in days), [Cr]_t=0_ is the creatine concentration before creatine withdrawal and k, the constant rate of creatine breakdown). Ninety days after creatine withdrawal, cardiac function was evaluated invasively by LV cannulation. A separate cohort of 6 AGAT^-/-^ fed creatine throughout the experiment was used as a control group for haemodynamic measurements.

***In vivo* LV haemodynamics**

Mice were anaesthetised with isoflurane in 100% medical oxygen and placed in a supine position on a homeothermic blanket (Harvard Apparatus). The right carotid artery was cannulated with a 1F mikro-tip catheter (Millar, Texas, USA) and advanced retrogradely across the aortic valve into the left ventricle. The jugular vein was cannulated with flame-stretched polyethylene cannula for the infusion of dobutamine (16ng/g BWt/min). Animals were allowed to equilibrate for at least 15 min before measurements under baseline conditions and steady-state dobutamine infusion. Mice were killed at the end of the experiments and their organs removed, washed in heparinised saline, blotted and weighed.

**High-energy phosphates**

Isolated perfused heart experiments were performed as previously described[^3^](#_ENREF_3) (constant pressure of 80 mmHg, 37°C) using Krebs-Henseleit (KH) buffer containing (mM) 11 D-glucose, 1.8 pyruvate and 0.2 lactate as substrates. ^31^P-MR-spectra were acquired using a pulse-and-collect sequence at a TR of 2s and a flip angle of 60° and 750 averages and were corrected for partial saturation. Thereafter, hearts were freeze clamped and total creatine and ATP content was measured by HPLC. To calculate the intracellular concentrations, we assumed total heart protein content to be 0.17g protein per gram wet weight and intracellular volume to be 0.5ml/gram wet weight.[^4^](#_ENREF_4) PCr, ATP and Pi concentrations were calculated by calibrating ^31^P MRS data to the ATP concentration in control hearts measured with HPLC. Plasma homoarginine levels were quantified in mouse plasma using an in-house stable isotope dilution assay for LC–MS/MS as described previously.[^5^](#_ENREF_5)

**Solution-state ^1^H-NMR spectroscopy**

LV tissue (~50 mg) from creatine-naïve mice (3 AGAT^-/-^ and 3 WT, age-matched females, mean 30 weeks) was analysed as previously described.[^6^](#_ENREF_6) Briefly, after methanol/chloroform extraction, the aqueous layer was dehydrated then rehydrated in D_2_O. Samples were analyzed using an Inova spectrometer operating at 400.13 MHz (Varian) and a solvent suppression pulse sequence based on a one-dimensional nuclear Overhauser effect spectroscopy pulse sequence to saturate the residual ^1^H water proton signal (relaxation delay = 2 s, *t*_1_= 3 µs, mixing time = 150 ms, solvent pre-saturation applied during the relaxation time and the mixing time).

**Enzyme activities and AMPK activation**

Total citrate synthase, and creatine kinase (CK) activity, and CK isoenzyme composition were measured as previously described.[^4^](#_ENREF_4) Adenylate kinase (AK) activity was measured in LV homogenates as described previously.[^7^](#_ENREF_7) AMPK activation was tested by immunoblotting as described previously[^8^](#_ENREF_8) using primary antibodies (Cell Signaling Technologies) against total AMPKα and phospho AMPKα (antibody detects endogenous AMPKα only when phosphorylated at threonine 172). Following quantification of chemiluminescent signal using Chemi-Doc imaging system and Image Lab software (Bio-Rad), AMPK activation was calculated by the ratio of phospho/total AMPKα expression.

**Isolated perfused heart function**

Creatine naïve AGAT^-/-^ (n=8) and WT (n=7) mice were anaesthetised with sodium pentobarbital (55 mg/kg I.P.) and heparin (300 IU). Hearts were rapidly excised, cannulated and perfused in Langendorff constant pressure mode at 80mmHg with oxygenated (95% O_2_/5% CO_2_) Krebs-Henseleit buffer at 37°C (mM): NaCl 118, KCl 4.7, MgSO_4_.7H_2_O 1.2, NaHCO_3_ 25, KH_2_PO_4_ 1.2, Glucose 11, CaCl_2_.H_2_O 1.8. LV function was assessed in spontaneously beating hearts using a water-filled intraventricular balloon connected to a pressure transducer (ADInstruments Ltd). The left ventricular end-diastolic pressure (LVEDP) was set to 6.1 ± 0.7 mmHg, and heart rate (HR) and left ventricular systolic pressure (LVSP) measurements collected. These parameters were used to calculate left ventricular developed pressure (LVDP = LVSP - LVEDP) and subsequently rate pressure product (RPP = HR * LVDP). Baseline function was continually recorded for 15 minutes. The ventricles were subsequently homogenised and mitochondria isolated for respiration experiments described below.

**Isolated cardiomyocyte function**

Left ventricular cardiomyocytes were isolated from WT and AGAT^-/-^ mice that had been fed a creatine containing diet throughout life. The heart was perfused with oxygenated Ca^2+^-free isolation solution (37°C) for 3 min and then with 2.5 mg/ml Liberase TH solution (Roche Diagnostics) for a further 7-10 min (until flow increased by 50%). The cell suspension was filtered through gauze and 1% BSA was added to terminate Liberase action. After centrifugation (500 rpm for 3 min) pellets were re-suspended with 1% BSA containing 500 µM Ca^2+^. Cells were left to settle at room temperature for 7 min before replacing the supernatant with 1% BSA containing 1 mM Ca^2+^. Cells were left to settle at room temperature for 30 min before starting single cell measurements. Cell shortening and re-lengthening velocity were measured in cardiomyocytes under field-stimulation at 3 Hz by using a video-edge detection system (IonOptix Corp). The [Ca^2+^]i transient was measured in fura-2 loaded (5 µmol/L, Molecular Probes) myocytes field-stimulated at 3 Hz. The amplitude of the [Ca^2+^]i transient was calculated as the difference between diastolic and peak Ca^2+^ fluorescence. The rate of decay of the field-stimulated [Ca2+]i transient was best fit by an exponential curve (Clampfit, Axonpatch, Axon Instruments) and tau used for comparisons between WT and AGAT^-/-^. A total of n=52/54 cells from 6/6 hearts per genotype were analysed.

**Mitochondrial respiration**

Cardiac mitochondria were isolated from creatine naïve AGAT^-/-^ (n=8) and WT mice (n=7) according to Rosca *et al.* (2008), except for some modifications.[^9^](#_ENREF_9) Briefly, hearts were rapidly excised and washed in ice-cold buffer. The LV was weighed and finely sliced into small sections in a Petri dish containing Chappel-Perry (CP) buffer (100mM KCl, 50mM MOPS, 5.0mM MgSO_4_, 1.0mM EGTA, 1.0mM ATP, pH 7.4 at 4°C). The heart was transferred to a glass Potter–Elvehjem homogeniser containing 100µl CP buffer/10mg of ventricular tissue and gently homogenised using a tapered bottom PTFE pestle. The homogenate was supplemented with 10% 2.5mg/ml trypsin solution and incubated for 10 min at 4°C. The activity of trypsin was attenuated with an equal volume of CP buffer plus 2mg/ml fatty acid free BSA. Mitochondria were isolated by differential centrifugation including a slow spin at 900 g to pellet myofibrillar components, followed by centrifugation of the supernatant at 5200 g to pellet the mitochondrial fraction. Mitochondria were washed twice and suspended in KME (100 mM KCl, 50 mM MOPS, and 0.5 mM EGTA, pH 7.4), kept on ice and used within 3 hours of isolation). Mitochondrial protein concentration was determined by the bicinchoninic acid (BCA) method.

Basal respiration was assessed with a Clark-type electrode using the Mitocell S200A Micro Respiratory system (Strathkelvin Instruments, Motherwell, UK). Isolated mitochondria (300µg) were equilibrated in the Mitocell chamber at a final volume of 0.3ml respiration medium containing 0.5mM EGTA, 3mM MgCl_2_6H_2_O, 60mM K-lactobionate, 20mM taurine, 10mM KH_2_PO_4_, 20mM HEPES, 110mM sucrose and 1g/l fatty acid free BSA, pH7.4 at 25°C. Basal respiration was initiated by the addition of glutamate (5mM), malate (2.5mM) and Na^+^-pyruvate (5mM) as substrates. Sequential additions of 150µM ADP, 2µM oligomycin and 100nM repeated doses of carbonyl cyanide-*4*-(trifluoromethoxy)phenylhydrazone (FCCP) examined state 3, state 4 and uncoupled respiration, respectively. Non-mitochondrial respiration was determined at the end of the experiment by addition of antimycin A (0.25µg/ml). Respiratory control ratios (RCR, state3: state4) were calculated to determine mitochondrial integrity or coupling.

**qRT-PCR**

Total RNA was extracted from ~ 10mg left ventricular tissue using Trizol reagent (Invitrogen) and further purified using RNeasy Mini Kit (Qiagen). Real-time PCR was performed using Quantitect SYBR Green RT-PCR Kit (Qiagen) in a Rotor-gene 3000 (Corbett Research/Qiagen). The levels of expression of all transcripts were normalized to 36B4 using the 2^-∆∆CT^ method[^10^](#_ENREF_10) and expressed as percentage of controls. The primers sequence were as follows: ANP 5’-GTGTACAGTGCGGTGTCCAA-3’ (F) and 5’-ACCTCATCTTCTACCGGCATC-3’ (R);[^11^](#_ENREF_11) BNP 5’-GAGGTCACTCCTATCCTCTGG-3’ (F) and 5’-GCCATTTCCTCCGACTTTTCTC-3’ (R);[^12^](#_ENREF_12) β-MHC 5’-GCATTCTCCTGCTGTTTCCTT-3’ (F) and 5’-TGGATTCTCAAACGTGTCTAGTGA-3’ (R);[^11^](#_ENREF_11) α-SA 5’-CCCAAAGCTAACCGGGAGAAG-3’ (F) and 5’-CCAGAATCCAACACGATGCC-3’ (R);[^12^](#_ENREF_12) CrT 5’-TTCAACAACAACTGCTACAAGG-3’ (F) and 5’-ATGAAGCCCAGGATGGAGAA-3’ (R); 36B4 5’-AGATTCGGGATATGCTGTTGGC -3’ (F) and 5’-TCGGGTCCTAGACCAGTGTTC -3’ (R).[^12^](#_ENREF_12)

**Cardiomyocyte cross-sectional area**

Hearts obtained from 3 WT and 3 AGAT^-/-^ creatine-naïve mice were fixed in 10% buffered formalin, dehydrated, and embedded in paraffin. Eight-micron-thick sections were prepared from the apex to the base. Sections were stained with Masson’s trichrome and examined under a light microscope. Images of 6 segments per heart were taken using a Nikon TE2000U microscope at 60x oil objective and 1.0x optical 110 zoom. Two random fields from each of the 6 segments were analysed equating to measurements on ~150-200 myocytes per animal. Quantification of the myocyte cross-sectional area was performed using Image J software version 1.46r (National Institutes of Health).

***In vivo* MR experiments**

All *in vivo* MR experiments were carried out under isoflurane anaesthesia on a 9.4 T (400 MHz) MR system (Agilent Technologies) and using a quadrature-driven birdcage resonator (Rapid Biomedical).

High-resolution murine Magnetic Resonance cine Imaging (cine MRI) was performed *in vivo* to assess LV mass and volumes, as previously described.[^13^](#_ENREF_13) Seven to eight short-axis slices covering the heart from base to apex were acquired using a cardiac-triggered and respiration-gated fast low-angle-shot sequence. The imaging parameters were: field of view (25.6 mm)^2^, matrix size 128 x 128, echo time (TE) / repetition time (TR) = 1.79/4.6 ms, 15° sinc excitation pulse, number of averages (NA) = 1.

Cardiac ^1^H-MRS was performed as previously described.[^14^](#_ENREF_14) Briefly, three water suppressed (NA = 256, on-resonance on creatine) and three non-water-suppressed scans (NA=16, on-resonance on water) were acquired interleaved during diastole from a 2 μL septal voxel using a cardiac-triggered / respiratory-gated PRESS sequence (TE/TR = 8 ms / 2 s). Spectra were quantified using AMARES algorithm from the jMRUI software.[^15^](#_ENREF_15) Creatine peak amplitudes were normalized to the amplitude of the water peak of the non-suppressed spectra acquired immediately before. Myocardial creatine concentration was then estimated using a calibration curve validated against HPLC measurements.

**Supplementary Tables**

**Supplementary Table S1:** Cardiac function in AGAT^-/-^ mice after dietary creatine withdrawal.

|  | **AGAT^-/-^ Cr-fed controls** | **AGAT^-/-^ before and after**  **Cr-withdrawal** | |
| --- | --- | --- | --- |
| *Cine-MRI (83 days)* | **n=6** | **n=6** | **n=6** |
| LV mass (mg) | 85 ± 4 | 81 ± 11 | 74 ± 10 ** |
| Heart rate (bpm) | 438 ± 9 | 454 ± 47 | 426 ± 26 |
| Ejection fraction (%) | 67 ± 7 | 70 ± 5 | 73 ± 4 |
| End-diastolic volume (μL) | 51 ± 12 | 48 ± 4 | 58 ± 7 |
| End-systolic volume (μL) | 16 ± 6 | 13 ± 2 | 19 ± 6 |
| Stroke volume (μL) | 39 ± 6 | 35 ± 7 | 35 ± 3 |
| Cardiac output (mL/min) | 17 ± 2 | 16 ± 3 | 16 ± 3 |
| *Post-mortem (90 days)* |  |  |  |
| Body weight (g) | 24 ± 3 | 23 ± 3 | 20 ± 3 * |
| LV weight (mg) | 87 ± 7 |  | 72 ± 10 * |
| Lung weight (mg) | 137 ± 6 |  | 122 ± 10 * |
| Tibial length (mm) | 17.9 ± 0.4 |  | 17.8 ± 0.3 |
| LV wt / BW (mg/g) | 3.6 ± 0.2 |  | 3.7 ± 0.3 |
| LV wt / tibial length (mg/mm) | 4.0 ± 0.5 |  | 4.8 ± 0.3 ** |

Data is mean ± SD, * denotes P<0.05, ** P<0.01.

**Supplementary Table S2:** Haemodynamic parameters in AGAT^+/+^ control mice fed a creatine-free diet (WT), 1 week or 7 weeks of creatine (Cr) or homoarginine (HA) supplementation.

|  | **WT control**  *(n = 10)* | **1 wk Cr – WT**  *(n = 7)* | **7 wk Cr – WT**  *(n = 8)* | **HA – WT**  *(n = 4)* |
| --- | --- | --- | --- | --- |
| Heart rate (bpm) | 451 ± 12 | 451 ± 7 | 447 ± 22 | 476 ± 23 |
| LV systolic pressure (mmHg) | 101 ± 2 | 105 ± 2 | 102 ± 2 | 103 ± 2 |
| LV diastolic pressure (mmHg) | 4.1 ± 0.8 | 7.3 ± 0.8 | 6.0 ± 0.9 | 6.4 ± 0.6 |
| dP/dt_max_ (mmHg/s) | 8732 ± 715 | 7943 ± 610 | 9116 ± 624 | 9623 ± 709 |
| dP/dt_min_ (mmHg/s) | -7518 ± 896 | -6313 ± 963 | -8067 ± 750 | -8844 ± 878 |

Values are mean ± SEM. There were no significant differences between groups using one-way ANOVA and Dunnett's Multiple Comparison Test for comparison to WT controls.

**Supplementary Table S3:** Statistical analysis of cardiomyocyte shortening and Ca^2+^ transient variables

|  |  | **t-test of**  **WT vs AGAT KO** | |  | **Hierarchical test of**  **WT vs AGAT KO** | | **Superior fit by clustering? (p-value)** |
| --- | --- | --- | --- | --- | --- | --- | --- |
|  | **Clustering of data (ICC) (%)** | **Std error of difference** | **p-value** |  | **Std error of difference** | **p-value** |  |
| Fractional Shortening (%) | 12.5% | 0.265 | 0.0085 |  | 0.445 | 0.0833 | Y (0.0038) |
| Shortening Velocity (μm/s) | 0% | 12.754 | 0.0317 |  | 12.691 | 0.0308 | N (1) |
| Re-lengthening Velocity (μm/s) | 0% | 12.722 | 0.0089 |  | 12.66 | 0.0086 | N (1) |
| Log Ca^2+^ Amplitude (F/F0) | 31.5% | 0.029 | 0.9203 |  | 0.053 | 0.8569 | Y (<0.0001) |
| Log Diastolic Ca^2+^ Ratio | 3.1% | 0.009 | 0.5088 |  | 0.01 | 0.5754 | N (0.5855) |
| Tau (ms) | 28.0% | 4.035 | 0.6222 |  | 7.104 | 0.9281 | Y (<0.0001) |

For each variable the degree of clustering was assessed using the intraclass correlation (ICC) and differences between WT and HA-deficient cardiomyocytes were assessed using a Student’s t-test and by a hierarchical test. Variables in which significant clustering was observed between isolations show a better goodness of fit. In all cases p-values from the hierarchical statistical analysis were used to reject the null hypothesis. For the Ca^2+^ transient amplitude and the diastolic Ca^2+^ ratio, data showed skewed symmetry which was adjusted using logarithmic transformation prior to statistical analysis.

**Supplementary Figure** Cardiac cine-MRI of wild-type (WT) and creatine-naïve AGAT^-/-^ (KO) mice at 21 weeks of age. (A) Representative end-diastolic short-axis images at mid-papillary level from WT (left) and KO (right) shown at the same scale. (B) Body weight, (C) LV mass calculated from MRI, (D) heart rate, (E) end-diastolic volume, (F) end-systolic volume, (G) ejection fraction, (H) stroke volume, (J) cardiac output. Both groups are n=10 consisting of 5 males and 5 females, and data are represented as mean ± SD.

**Supplemental References**

1. Choe C-u, Nabuurs C, Stockebrand MC, Neu A, Nunes P, Morellini F *et al.* l-arginine:glycine amidinotransferase deficiency protects from metabolic syndrome. *Hum Mol Genet* 2013;**22**:110-123.

2. Kingsbury M, Mahnke A, Turner M, Sheridan D. Recovery of coronary function and morphology during regression of left ventricular hypertrophy. *Cardiovasc Res* 2002;**55**:83-96.

3. ten Hove M, Lygate CA, Fischer A, Schneider JE, Sang AE, Hulbert K *et al.* Reduced inotropic reserve and increased susceptibility to cardiac ischemia/reperfusion injury in phosphocreatine-deficient guanidinoacetate-N-methyltransferase-knockout mice. *Circulation* 2005;**111**:2477-2485.

4. Neubauer S, Horn M, Naumann A, Tian R, Hu K, Laser M *et al.* Impairment of energy metabolism in intact residual myocardium of rat hearts with chronic myocardial infarction. *J Clin Invest* 1995;**95**:1092-1100.

5. Cordts K, Atzler D, Qaderi V, Sydow K, Böger RH, Choe C-u *et al.* Measurement of homoarginine in human and mouse plasma by LC–MS/MS and ELISA: a comparison and a biological application. *Amino Acids* 2015;**47**:2015-2022.

6. Atherton HJ, Bailey NJ, Zhang W, Taylor J, Major H, Shockcor J *et al.* A combined 1H-NMR spectroscopy- and mass spectrometry-based metabolomic study of the PPAR-alpha null mutant mouse defines profound systemic changes in metabolism linked to the metabolic syndrome. *Physiol Genomics* 2006;**27**:178-186.

7. Aksentijevic D, Lygate CA, Makinen K, Zervou S, Sebag-Montefiore L, Medway D *et al.* High-energy phosphotransfer in the failing mouse heart: role of adenylate kinase and glycolytic enzymes. *Eur J Heart Fail* 2010;**12**:1282-1289.

8. Zervou S, Ray T, Sahgal N, Sebag-Montefiore L, Cross R, Medway DJ *et al.* A role for thioredoxin-interacting protein (Txnip) in cellular creatine homeostasis. *Am J Physiol Endocrinol Metabol* 2013;**305**:E263-270.

9. Rosca MG, Vazquez EJ, Kerner J, Parland W, Chandler MP, Stanley W *et al.* Cardiac mitochondria in heart failure: decrease in respirasomes and oxidative phosphorylation. *Cardiovasc Res* 2008;**80**:30-39.

10. Livak KJ, Schmittgen TD. Analysis of relative gene expression data using real-time quantitative PCR and the 2(-Delta Delta C(T)) Method. *Methods* 2001;**25**:402-408.

11. Gaussin V, Tomlinson JE, Depre C, Engelhardt S, Antos CL, Takagi G *et al.* Common genomic response in different mouse models of beta-adrenergic-induced cardiomyopathy. *Circulation* 2003;**108**:2926-2933.

12. Wang X, Seed B. A PCR primer bank for quantitative gene expression analysis. *Nucleic Acids Res* 2003;**31**:e154.

13. Schneider JE, Wiesmann F, Lygate CA, Neubauer S. How to perform an accurate assessment of cardiac function in mice using high-resolution magnetic resonance imaging. *J Cardiovasc Magn Reson* 2006;**8**:693-701.

14. Schneider JE, Tyler DJ, ten Hove M, Sang AE, Cassidy PJ, Fischer A *et al.* In vivo cardiac 1H-MRS in the mouse. *Magn Reson Med* 2004;**52**:1029-1035.

15. Vanhamme L, van den Boogaart A, Van Huffel S. Improved method for accurate and efficient quantification of MRS data with use of prior knowledge. *J Magn Reson* 1997;**129**:35-43.
